# Supplementary material for: Quantitative analysis of plant ER architecture and dynamics
Source: Nat Commun. 2019 Feb 28;10:984. doi: 10.1038/s41467-019-08893-9 (PMC6395764; doi:10.1038/s41467-019-08893-9)
Supplement: Supplementary file 1 — Supplementary Information [file 41467_2019_8893_MOESM1_ESM.pdf]

# Quantitative analysis of plant ER architecture and dynamics

Pain *et al.*

Supplementary Information

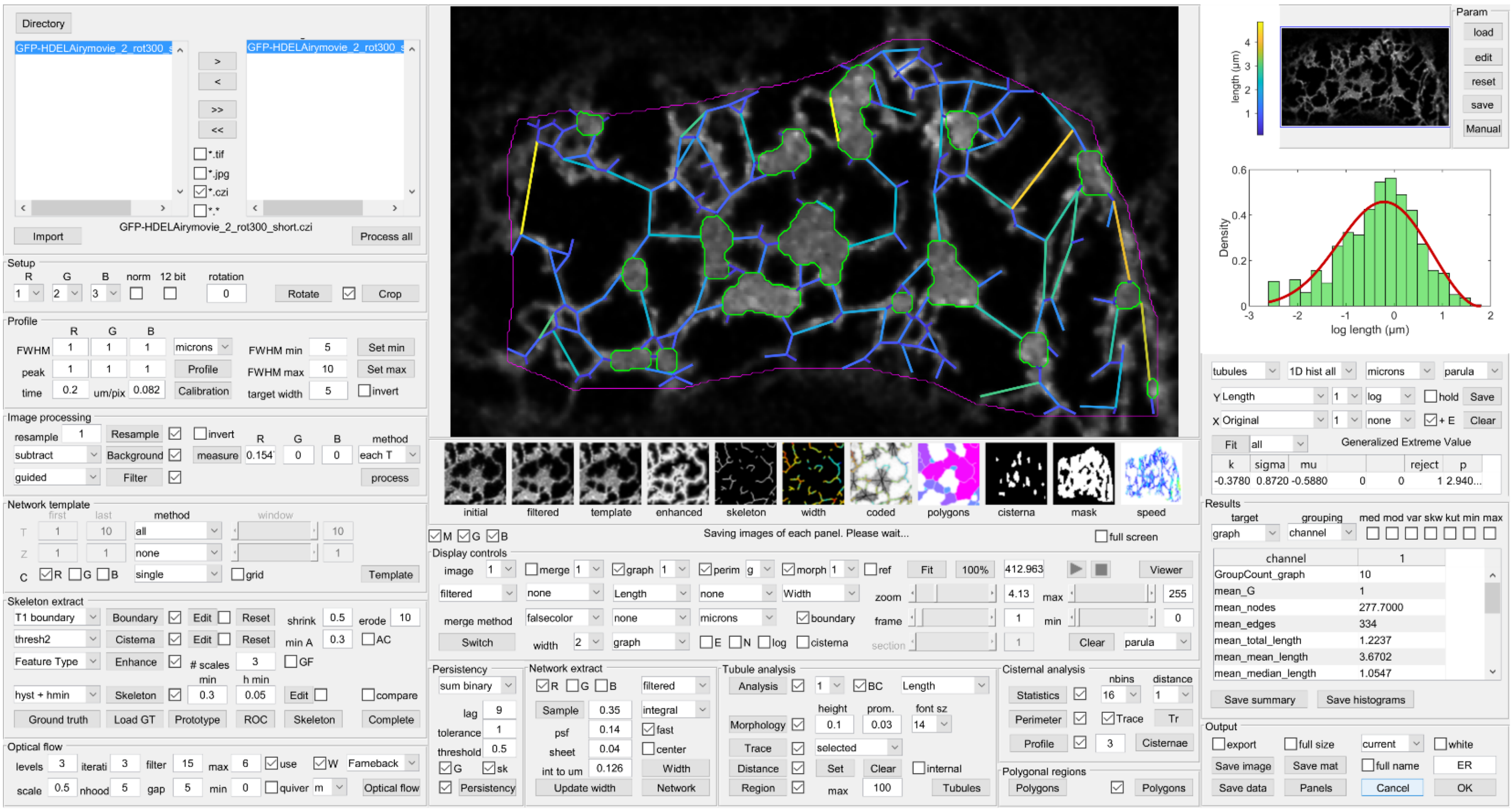

**Supplementary figure 1: AnalyzER: A Graphical User Interface (GUI) for rapid ER analysis**
The GUI used for complete analysis of ER structure and dynamics, demonstrating typical settings for the analysis of a 50 frame, single channel time series, with the graph representation of tubule length and cisternal area shown. A complete description of the interface is available in the accompanying manual available from the Oxford Research Archive: https://ora.ox.ac.uk/objects/uuid:cb0e2845-2a9c-495a-84f0-4dd2c5164463

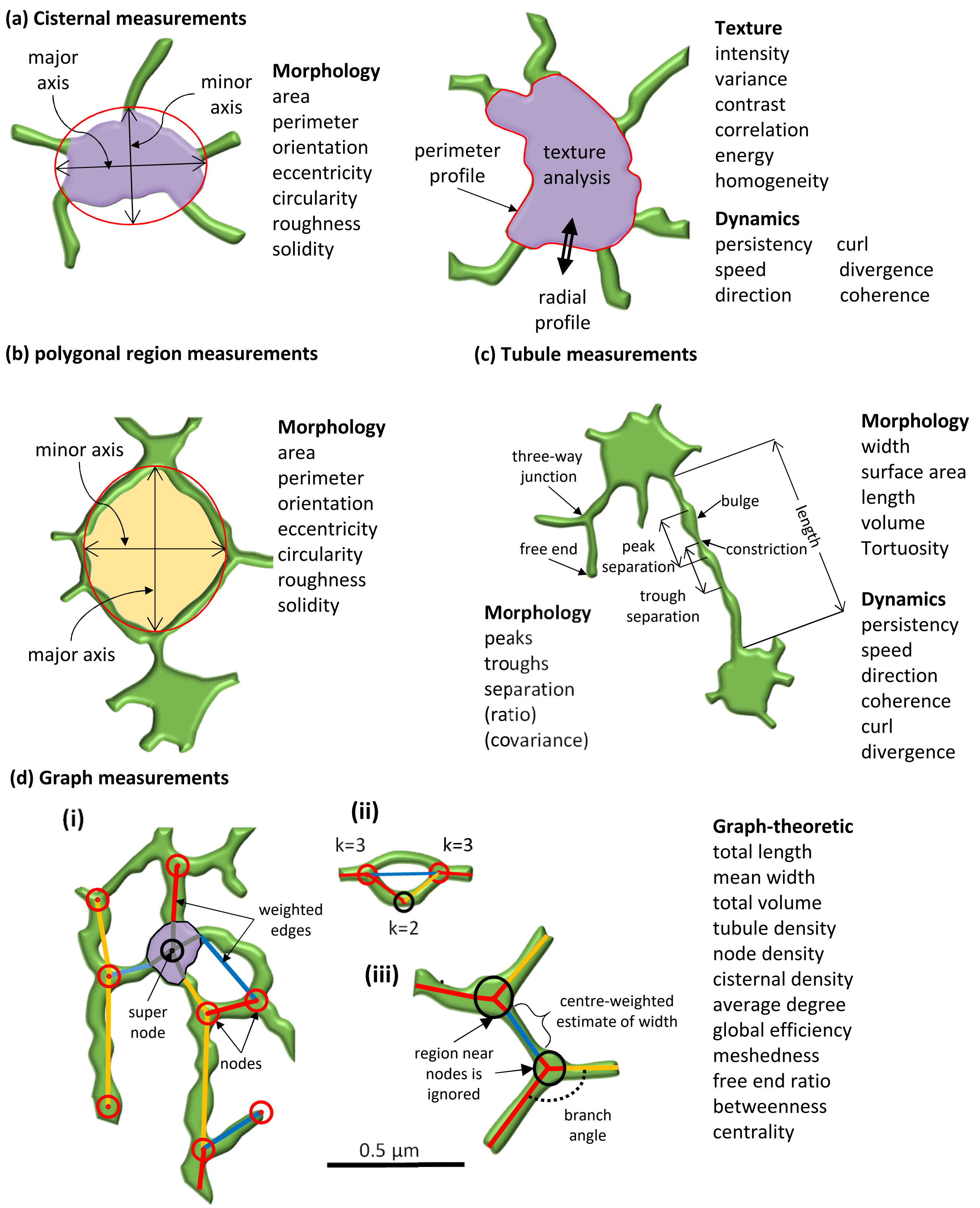


**Supplementary figure 2: Schematic illustrations of the main ER metrics automatically measured in the AnalyzER program**

Schematic diagrams showing the morphological measurements made for cisternae (a); polygonal regions (b); and tubules (c); along with the conversion to a weighted graph representation where each edge and node was associated with a vector of properties (d(i)). (d(ii)) Two tubules connected to the same junctions were resolved in the graph representation by creating an additional k=2 node in the longer arm. (d(iii)) The tubule width was estimated from the central part of the tubule excluding the region near the nodes.

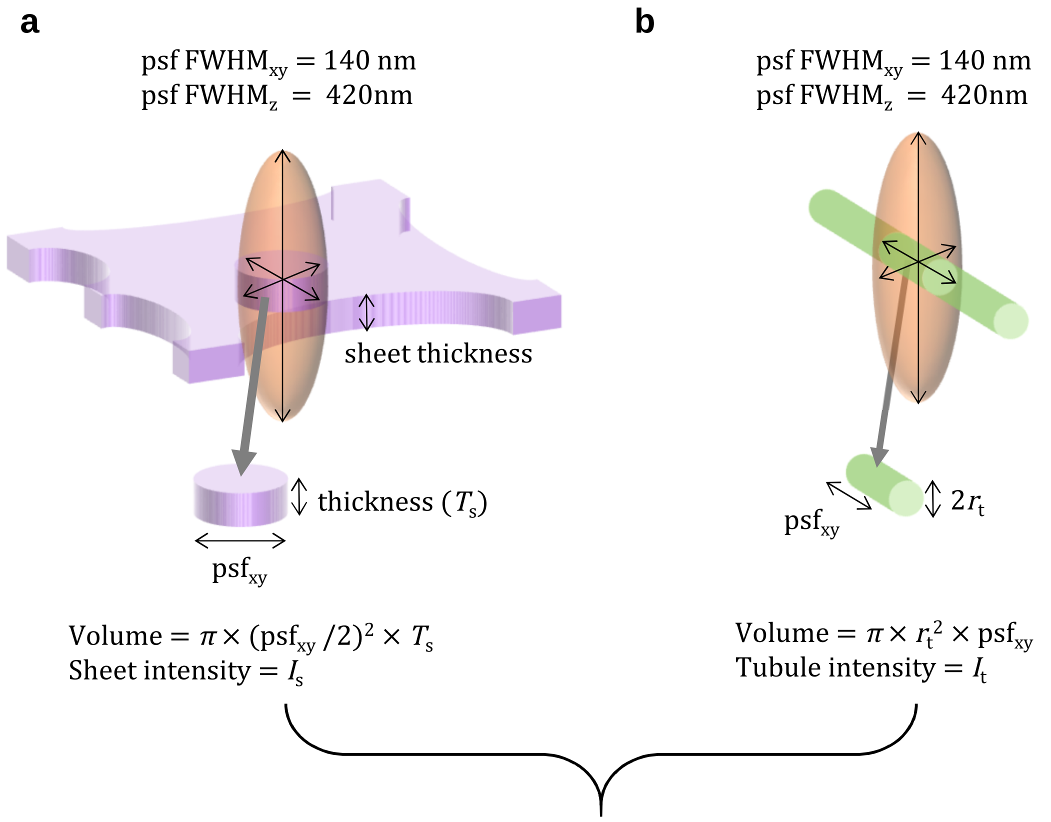


$$r_t = \sqrt{\frac{I_t \times \text{psf}_{xy} \times T_s}{I_s \times 4}}$$

**Supplementary figure 3: Estimation of tubule width from intensity measurements**

(a) A cisternal sheet provides an internal reference for the amount of fluorescence intensity ($I_s$) expected from a defined volume of ER when sampled with a stylised point spread function (psf), shown here with dimensions appropriate for the Zeiss Airyscan confocal microscope. The volume approximates to a flattened cylinder with a radius given by half the full-width half-maximum (FWHM) of the psf in the xy plane (psf$_{xy}$), and the length equivalent to the thickness of the sheet ($T_s$), independently measured as 40.51 nm ± 0.82 (mean ± SD, n=1, 25 technical repeats) using SBF-SEM. (b) The intensity ($I_t$) for a tubule sampled with the same psf would be expected to scale with the relative volume of the tubule, approximated as a horizontal cylinder with length equal to psf$_{xy}$ and the radius ($r_t$) as the unknown. The radius is estimated as: $r_t = \sqrt{\frac{I_t \times \text{psf}_{xy} \times T_s}{I_s \times 4}}$

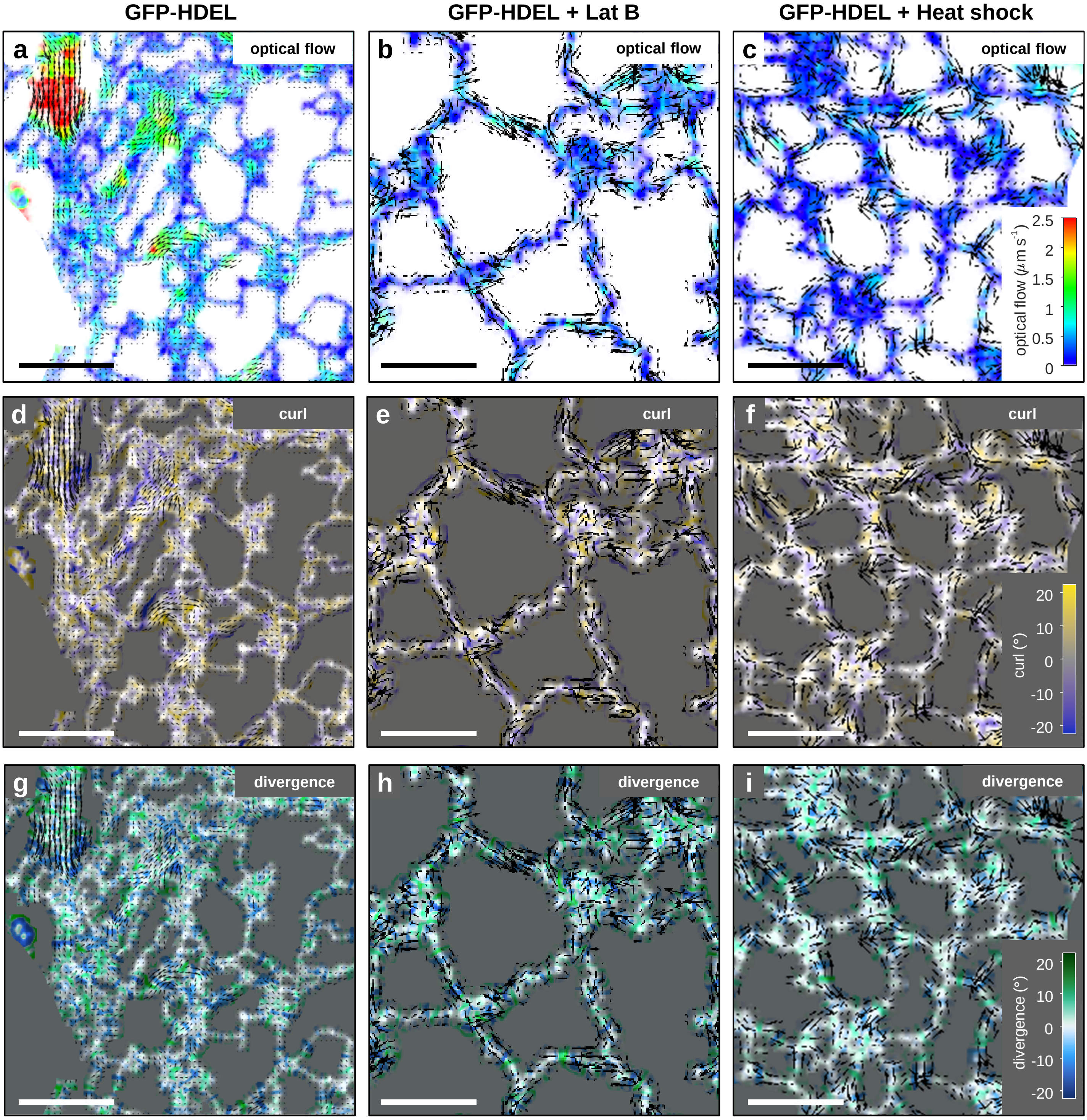


**Supplementary Figure 4: Mapping flow curl and divergence**
The velocity field calculated from the optical flow (a-c) was analysed for the local curl (d-f) and divergence (g-i) for HDEL controls (a,d,g), and after treatment with Lat B (b,e,h) or heat shock (c,f,i). Images were pseudo-colour coded according to the inset colour scales. Scale bars = 5 µm.

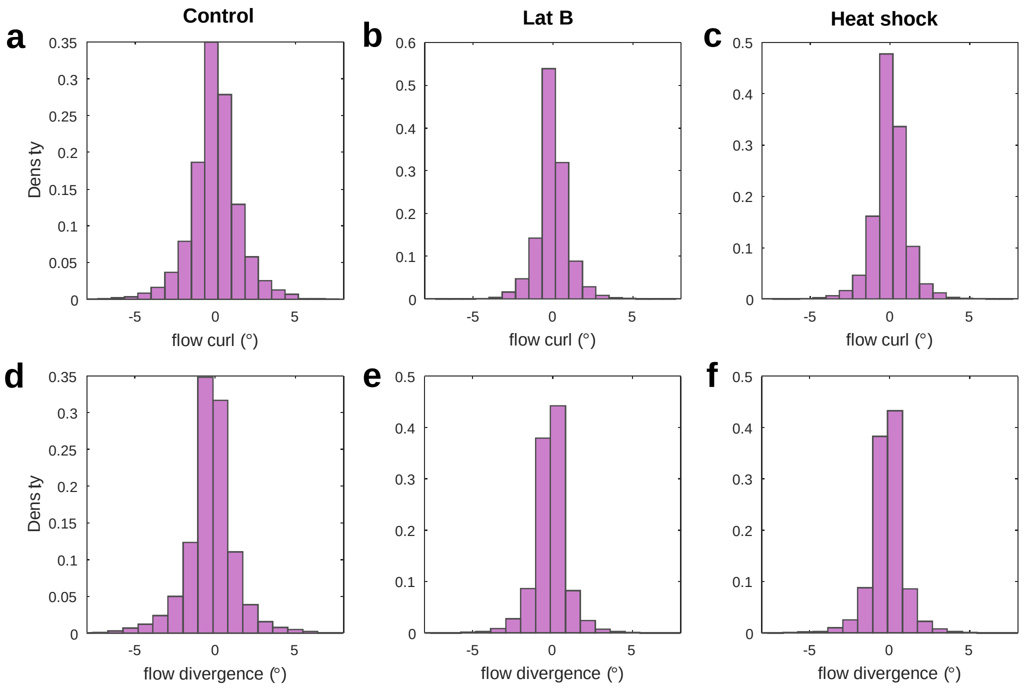


**Supplementary figure 5: Quantifying flow curl and flow divergence for cisternae**

Density histograms of flow curl (a-c) and flow divergence (d-f) derived from optical flow measurements of cisternae from HDEL controls (a,d), compared to Lat B (b,e) or heat shock (c,f) show a greater spread in the distribution for the controls for both variables, but little difference between Lat B and heat shock treatments (GFP-HDEL, n=6, Lat B, n=8, Heat shock, n=8, where n is the number of independent 50-frame time-series images). Source data are provided as a Source Data file.

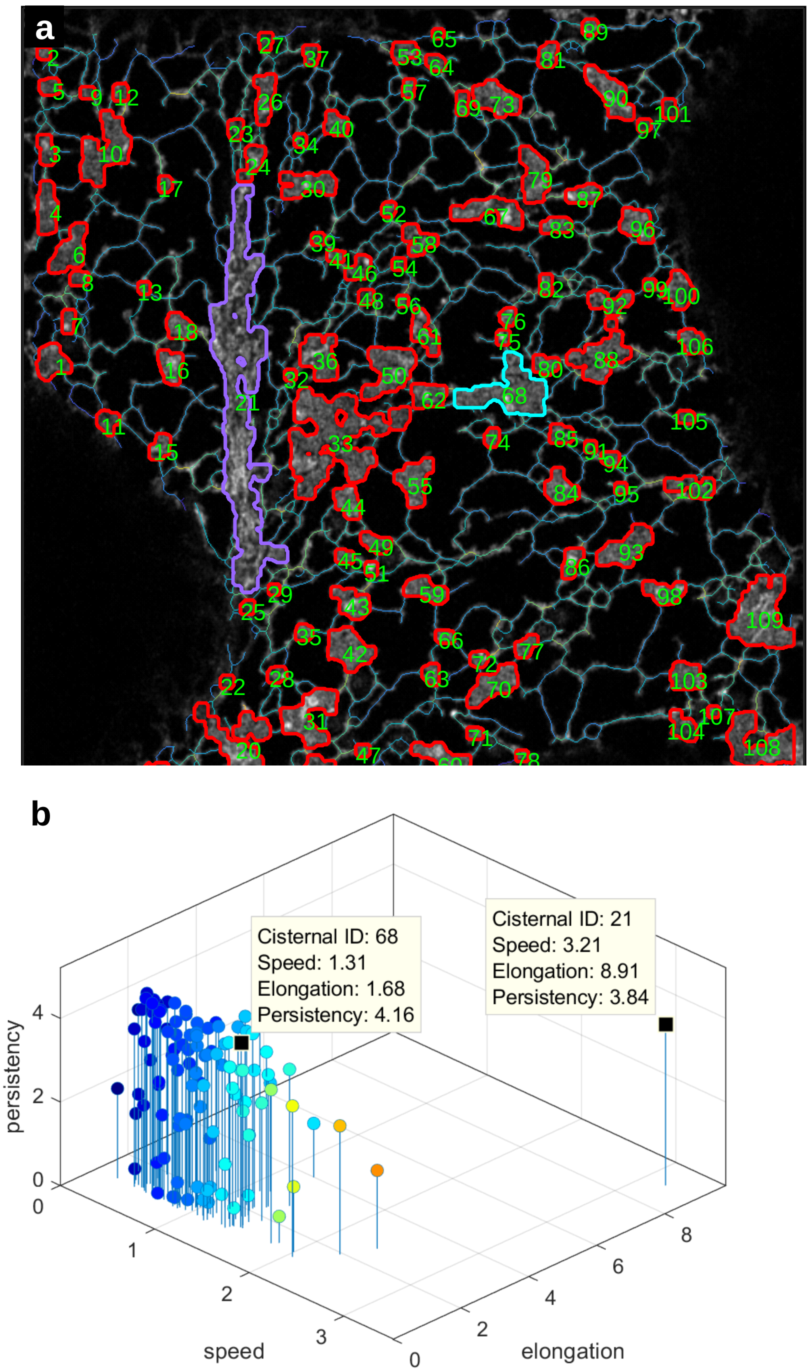


**Supplementary figure 6: Classification of cisterna**

Cisternae are identified as structures remaining after removal of the tubules by image opening. These structures include sheet-like regions of the ER, but also regions where tubules are closely appressed that cannot be segmented without recourse to super-resolution techniques. This is particularly true in the streaming cytoplasm, where segmentation is further complicated by movement. However, it is straightforward to group cisternae into different classes using a range of metrics. (a) shows a single image from a 50 frame time series of a tobacco epidermal cell expressing GFP-HDEL, with the tubule pixel skeleton colour-coded according to average width, and the cisternae outlined in red. An elongated cisterna in the streaming cytoplasm that probably represents a ribbon of appressed tubules and cisternae that cannot be resolved, is outlined in magenta, and a reference cisterna in cyan. (b) a 3D scatter plot of all the cisternae shows the cisterna in the streaming cytoplasm is clearly separated from the other cisternae in terms of elongation and speed.
