# Supplementary material for: Quantitative analysis of plant ER architecture and dynamics
Source: Nat Commun. 2019 Feb 28;10:984. doi: 10.1038/s41467-019-08893-9 (PMC6395764; doi:10.1038/s41467-019-08893-9)
Supplement: Supplementary file 2 — Description of Additional Supplementary Files [file 41467_2019_8893_MOESM2_ESM.docx]

**Description of Additional Supplementary Files**

File Name: Supplementary Movie 1

Description: Measurement of ER dynamics.
