# Supplementary material for: Quantitative analysis of plant ER architecture and dynamics
Source: Nat Commun. 2019 Feb 28;10:984. doi: 10.1038/s41467-019-08893-9 (PMC6395764; doi:10.1038/s41467-019-08893-9)
Supplement: Supplementary file 5 — Reporting Summary [file 41467_2019_8893_MOESM5_ESM.pdf]

nature research

Corresponding author(s): M.D. Fricker

Reporting Summary

Nature Research wishes to improve the reproducibility of the work that we publish. This form provides structure for consistency and transparency in reporting. For further information on Nature Research policies, see Authors & Referees and the Editorial Policy Checklist.

Statistical parameters

When statistical analyses are reported, confirm that the following items are present in the relevant location (e.g. figure legend, table legend, main text, or Methods section).

n/a	Confirmed	
☐	☒	The exact sample size (*n*) for each experimental group/condition, given as a discrete number and unit of measurement
☐	☒	An indication of whether measurements were taken from distinct samples or whether the same sample was measured repeatedly
☐	☒	The statistical test(s) used AND whether they are one- or two-sided<br>*Only common tests should be described solely by name; describe more complex techniques in the Methods section.*
☒	☐	A description of all covariates tested
☐	☒	A description of any assumptions or corrections, such as tests of normality and adjustment for multiple comparisons
☐	☒	A full description of the statistics including central tendency (e.g. means) or other basic estimates (e.g. regression coefficient) AND variation (e.g. standard deviation) or associated estimates of uncertainty (e.g. confidence intervals)
☐	☒	For null hypothesis testing, the test statistic (e.g. *F*, *t*, *r*) with confidence intervals, effect sizes, degrees of freedom and *P* value noted<br>*Give P values as exact values whenever suitable.*
☒	☐	For Bayesian analysis, information on the choice of priors and Markov chain Monte Carlo settings
☒	☐	For hierarchical and complex designs, identification of the appropriate level for tests and full reporting of outcomes
☒	☐	Estimates of effect sizes (e.g. Cohen's *d*, Pearson's *r*), indicating how they were calculated
☐	☒	Clearly defined error bars<br>*State explicitly what error bars represent (e.g. SD, SE, CI)*

Our web collection on statistics for biologists may be useful.

Software and code

Policy information about availability of computer code

Data collection	Zeiss Zen software was used for all confocal data collection.<br>SBF-SEM images were collected with the Gatan 3View software, aligned with the IMOD package and analysed with Amira
Data analysis	All image processing and data analysis was performed with a custom MatLab software package. A compiled Windows version of the software, and a MatLab app that installs the source code are freely available on the Oxford Research Archive. with a permanent URL. There is also a 120 page manual, a tutorial and test data sets.<br>All statistical analysis used standard MatLab MANOVA and ANOVA functions.

For manuscripts utilizing custom algorithms or software that are central to the research but not yet described in published literature, software must be made available to editors/reviewers upon request. We strongly encourage code deposition in a community repository (e.g. GitHub). See the Nature Research guidelines for submitting code & software for further information.

Data

Policy information about availability of data

All manuscripts must include a data availability statement. This statement should provide the following information, where applicable:

- Accession codes, unique identifiers, or web links for publicly available datasets
- A list of figures that have associated raw data
- A description of any restrictions on data availability

All the image datasets analysed in the study, along with the processing parameters, have been deposited on the Oxford Research Archive.
A zipped source data file is included with the data used for the specific graphs shown in the figures and supplementary figures

Field-specific reporting

Please select the best fit for your research. If you are not sure, read the appropriate sections before making your selection.

☒ Life sciences ☐ Behavioural & social sciences ☐ Ecological, evolutionary & environmental sciences

For a reference copy of the document with all sections, see nature.com/authors/policies/ReportingSummary-flat.pdf

Life sciences study design

All studies must disclose on these points even when the disclosure is negative.

Sample size	The sample size for each treatment in main analysis was set to be at least 40 time-series images, which is 2-3x the norm for most publications on ER, in part to demonstrate the capacity of the software to handle medium-throughput image screening. In practice 325 time-series movies were collected to ensure sufficient n numbers after data exclusion (see below)
Data exclusions	Time-series were rejected prior to analysis if there was z-drift, the contiguous ER network was too small or fragmented, or the signal-to-noise was insufficient due to low construct expression. One batch had the wrong averaging setting so was not comparable. A complete spreadsheet of all images and the rejection criteria is included in the data archive.
Replication	The software was developed and tested on data from multiple pilot studies prior to the main data collection. Specifically:<br>Reticulon experiments on morphology: 3 independent pilot studies, data for fig 2 collected from two biological replicates (i.e. two plants).<br>Lunapark cisternal distribution: 2 pilot studies, data for fig 3 collected from two biological replicates (i.e. two plants).<br>Speed/persistency: Pilot studies performed within the ANOVA experiments. Single session used to capture the extended time series.<br>MANOVA experiment : 6 plants, 4 separate imaging sessions, random combinations of 2-3 treatments per session, plus a control.
Randomization	The main constraint for full randomisation is the time taken for imaging that limits the number of samples per session, typically 25-50 images per session depending on expression, the plant and the amount of time needed for drug treatments to work. We therefore aimed for 3-4 combinations in one session including a control.
Blinding	Blinding was not relevant as all images were processed automatically

Reporting for specific materials, systems and methods

Materials & experimental systems

n/a	Involved in the study
☒	☐ Unique biological materials
☒	☐ Antibodies
☒	☐ Eukaryotic cell lines
☒	☐ Palaeontology
☒	☐ Animals and other organisms
☒	☐ Human research participants

Methods

n/a	Involved in the study
☒	☐ ChIP-seq
☒	☐ Flow cytometry
☒	☐ MRI-based neuroimaging
